# Personality traits and cancer survival: a Danish cohort study

**DOI:** 10.1038/sj.bjc.6603244

**Published:** 2006-07-04

**Authors:** N Nakaya, P E Hansen, I R Schapiro, L F Eplov, K Saito-Nakaya, Y Uchitomi, C Johansen

**Affiliations:** 1 Psycho-Oncology Division, Research Center for Innovative Oncology, National Cancer Center Hospital East, Kashiwa, Japan; 2 Department of Psychosocial Cancer Research, Institute of Cancer Epidemiology, Danish Cancer Society, Copenhagen, Denmark; 3 Copenhagen County Research Centre for Prevention and Health, Glostrup University Hospital, Glostrup, Denmark

**Keywords:** cancer survival, Danish, extraversion, neuroticism, prospective cohort study

## Abstract

We conducted a population-based prospective cohort study in Denmark to investigate associations between the personality traits and cancer survival. Between 1976 and 1977, 1020 residents of the Copenhagen County completed a questionnaire eliciting information on personality traits and various health habits. The personality traits extraversion and neuroticism were measured using the short form of the Eysenck Personality Inventory. Follow-up in the Danish Cancer Registry for 1976–2002 revealed 189 incidents of primary cancer and follow-up for death from the date of the cancer diagnosis until 2005 revealed 82 deaths from all-cause in this group. A Cox proportional-hazards model was used to estimate the hazard ratios (HRs) of death from all-cause according to extraversion and neuroticism adjusting for potential confounding factors. A significant association was found between neuroticism and risk of death (HR, 2.3 (95% CI=1.1–4.7); Linear trend *P*=0.04) but not between extraversion and risk of death (HR, 0.9 (0.4–1.7); Linear trend *P*=0.34). Similar results were found when using cancer-related death. Stratification by gender revealed a strong positive association between neuroticism and the risk of death among women (Linear trend *P*=0.03). This study showed that neuroticism is positively associated with cancer survival. Further research on neuroticism and cancer survival is needed.

Psychological traits may alter immune and endocrine function and it has long been hypothesised that, through this pathway, personality traits may affect cancer incidence and survival (Spiegel and Kato, 1996; Kiecolt-Glaser and Glaser, 1999; Dalton *et al*, 2002). The results of our earlier prospective cohort studies, however, do not support the hypothesis that personality traits measured by the Eysenck questionnaire are associated with risk for cancer (Schapiro *et al*, 2001; Nakaya *et al*, 2003; Hansen *et al*, 2005).

The role of personality traits in cancer survival has been controversial. Five studies have investigated associations between personality traits and cancer survival. Two studies found statistically significant associations (Hislop *et al*, 1987; Ratcliffe *et al*, 1995) but three other studies found no association between personality traits and risk of death in cancer patients (Greer *et al*, 1979; Dean and Surtees, 1989; Nakaya *et al*, 2005). However, four studies had several limitations, including failure to control for cigarette smoking or alcohol consumption, and failing to assess personality traits before the cancer diagnosis (Greer *et al*, 1979; Hislop *et al*, 1987; Dean and Surtees, 1989; Ratcliffe *et al*, 1995).

We conducted a population-based prospective cohort study in Denmark to further investigate associations between the personality traits and cancer survival. This study focused on personality traits measured before the cancer diagnosis, and the study to our knowledge included the second highest number of cancer cases and deaths of the studies conducted until now.

## MATERIALS AND METHODS

### Study cohort

All persons born 1936 and living in four municipalities in a suburban area of Copenhagen in 1976 (*n*=1198 persons) were invited to participate in an epidemiologic health survey at the Glostrup Population Studies. The survey included a social-psychiatric interview (Hollnagel *et al*, 1981). Of these, 1052 persons (88%), equally distributed by sex, accepted the invitation. In a study, the study population was found to be representative of the age group 40 years in Copenhagen County (Hollnagel *et al*, 1981).

### Assessment of personality traits and other characteristics

The social-psychiatric interview lasted approximately 1 h and was performed by a physician. The questionnaire included marital status, socioeconomic status, smoking habits, alcohol consumption, personality, and the presence of symptoms of a psychiatric disease as evaluated by the interviewing doctor (Sælan and Garde, 1979). Personality traits were measured using a short form of the Eysenck Personality Inventory (EPI-Q) (Eysenck and Eysenck, 1965) consisting of 18 items (Floderus, 1974). This abbreviated version, the EPI-Q allows measurement of two dimensions: degree of extraversion and degree of neuroticism (Schapiro *et al*, 2001). Extraversion represents sociability, liveliness, and surgency and neuroticism represents emotional instability, and anxiousness (Eysenck and Eysenck, 1975).

### Linkage to registries

Data on all members of the study population were linked to the Central Population Register for verification of the personal identification number and for information on vital statistics and migration. The Central Population Register in Denmark was established on 1 April 1968, and all Danish residents are assigned a 10-digit personal identification number, which incorporates sex and date of birth and permits accurate linkage of information among registries. Subsequently, the study cohort was linked to the Danish Cancer Registry, which began reporting cancer incidence on a nationwide scale in 1943. Each record includes the personal identification number, date of diagnosis of the tumor, and information on the tumor. Tumors are coded according to a modified Danish version of the *International Classification of Diseases*, Seventh Revision (Jensen *et al*, 1985). Finally, the study cohort was linked to the Danish Registry of Causes of Death, which holds individual records of deaths in Denmark from 1943 and onwards (Juel and Helweg-Larsen, 1999). Cause of death was coded according to a modified Danish version of the *International Classification of Diseases*, Tenth Revision.

### Follow-up

From the 1052 persons in the study, we excluded 21 persons who had foreign citizenship as well as persons who had not completed the extraversion and neuroticism scales leaving 1020 persons. Members of the cohort were followed for cancer from the date of social-psychiatric interview, until the date of first cancer (other than non-melanoma skin cancer), date of emigration, date of death, or 31 December 2002. This resulted in a total of 189 cancer cases left for analysis and this subcohort was then followed-up for death from the date of the cancer diagnosis until date of emigration, date of death, or the end of the study period (31 January 2005). The 189 cancer cases (65 men and 124 women) of this study accrued approximately 1704 person-years of follow-up, with an average of 6 years. A total of 82 deaths from all causes (37 men and 45 women) were observed during follow-up. 84% of the deaths observed were cancer related, 16% were related to other causes. No deaths were classified as suicide.

### Statistical analysis

The objective of the current analyses was to determine the effect of extroversion and neuroticism on cancer survival. The personality subscales were included both as linear as well as ordinal variables. When using them as ordinal variables the scores on each personality subscale (score 0–9) were divided into three score levels approximately equal in size and as a result, the cutoff scores vary with the subscale. Hazard ratios (HRs) were computed as the death rate among subjects in each personality score level divided by the death rate among subjects in the lowest score level. A Cox proportional-hazard model was used to estimate HRs for extraversion and neuroticism. We used the SAS PHREG procedure in the SAS version 8.2 statistical software package (Cary, NC, USA) (Rothman and Greenland, 1998). All *P*-values were two-tailed. First, we tested which of a number of potential demographic and medical factors to include as confounding variables in the analyses. Then we performed univariate analyses (adjusted for sex and age at cancer diagnosis), as well as multivariate analyses (adjusted for potential confounders shown to have a statistically significant effect on survival). In addition to sex and age at cancer diagnosis (continuous variable), we considered the following variables as a priori confounders: cancer site (digestive organs, hormone-related organs, virus and immune-related organs, respiratory organs, or other organs) (World Health Organization, 1992; Schapiro *et al*, 2001); clinical stage (*in situ*, localised, regional invasion, distant metastasis, or unknown); length of education in years (<10, or >10); marital status (living with spouse, or living without spouse); social class (I–II, III–IV, or V); smoking status at baseline (never smokers, past smokers, or current smokers); alcohol consumption in grams of alcohol per week at baseline (0, 1–150 or 151 or more); and psychiatric status at time of interview (normal, neurotic, or deviant/psychotic). The social class variable was based on a classification constructed by the Social Research Institute in Denmark and is based on a combination of information on ownership of land, education, employment status, and number of subordinates. Social group I is the highest social group. The alcohol consumption variable was computed using the following definitions: one glass of beer or one glass of wine amounts to 12 g of alcohol, one glass of schnapps or other liqueur amounts to 9 g of alcohol. To test if there was a dose–response relationship between the personality subscales and cancer survival we did trend tests using the personality subscales as linear variables. We also investigated whether undiagnosed cancer could influence the personality scores by excluding persons diagnosed with cancer within the first 3 years. To investigate if other causes of death beside cancer could influence the results, analyses were performed on death from all causes as well as on death from cancer exclusively. We tested possible effect modifications of sex in order to investigate if personality traits have different effects among men and women. These tests of effect modification were performed by including an interaction term for sex and neuroticism in the analysis. We conducted stratified analyses according to 10-year age groups to investigate whether an effect of personality could be related to a specific age group. Finally, we did analyses where the personality scales were divided into four levels based on quartiles in order to test if changing the cutoff points would make a difference in the size of effect.

## RESULTS

First, we compared the characteristics of persons according to extraversion and neuroticism. Persons who scored high on extraversion were more likely to be male (*P*<0.01), current smokers at baseline (*P*=0.02), and consumers of alcohol at baseline (*P*<0.01). Persons who scored high on neuroticism were less likely to be male (*P*=0.03) and living with their spouse (*P*=0.01) and more likely to be described as neurotic in their psychiatric status (*P*<0.01) (Table 1).

In the univariate Cox proportional-hazard regression analyses, the following six of ten demographic and medical variables were significantly associated with cancer survival compared with each referent category (category that showed increased risk); age in years at cancer diagnosis (continuous variables), sex (man), cancer site (virus and immune-related organs or respiratory organs), clinical stage (*in situ*, regional invasion, distant metastasis, or unknown), smoking status (past smokers or current smokers), and alcohol consumption (⩾151 g week^−1^) (Table 2). Subjects described as neurotic in their psychiatric status showed an increased and borderline significant (*P*=0.053) risk of death from all causes compared to normal subjects. Being a theoretically interesting and borderline significant risk factor for death of all causes, we decided to include it in the further analyses.

The age and sex adjusted Cox proportional-hazards regression analysis showed no significant association between extraversion and the risk of death from all causes among persons diagnosed with cancer (Table 3). After controlling for age at cancer diagnosis, sex, cancer site, clinical stage, smoking status, and alcohol consumption at baseline (in multivariable HR1), the effect of extraversion on risk of death was basically unchanged (HR, 0.8 (95% confidence intervals (CI)=0.4–1.7); Linear trend *P*=0.33). Adding psychiatric status as a confounder in HR2 did not change the result (Linear trend *P*=0.34). The multivariable adjusted HR3 was then estimated excluding 19 persons who were diagnosed as having cancer within the first 3 years from the baseline, but no differences in effect were observed for extraversion. The result when looking exclusively at cancer-related death in HR4 did not show any significant effect of extroversion (HR, 0.6 (95% CI=0.3–1.3); Linear trend *P*=0.12).

For neuroticism and the risk of deaths from all causes among persons diagnosed with cancer, the age and sex adjusted HR showed no significant association. The multivariable adjusted HR (HR1) showed a significant association between neuroticism and the risk of death from all causes (HR, 2.6 (1.4–5.0); Linear trend *P*<0.01)). When adding psychiatric status as a confounder the association remained significant. The multivariable adjusted HR3 was estimated excluding persons diagnosed as having cancer within the first 3 years from the baseline, but only small differences in effect were observed (Linear trend *P*=0.02). When using cancer mortality as the end point (case=69) in HR4, a similar, significant, linear, positive association between neuroticism and the risk of death was observed (Linear trend *P*=0.04).

We furthermore conducted analyses stratified by sex. No significant association between extraversion and cancer survival was found among men and women. Among women, neuroticism was strongly associated with cancer survival in multivariable adjusted HR2 (Linear trend *P*=0.03), and the same pattern was observed among men although the estimates were not significant. Also, when including sex and neuroticism as an interaction term, the associations between neuroticism and the risk of death were not remarkably modified by sex (*P*>0.05). When stratifying according to 10-year age groups, cancer type, clinical stage, smoking, and alcohol consumption, the associations between neuroticism and the risk of cancer were not remarkably modified. Also, changing the cutoff points for the personality scales from tertiles to quartiles did not make any change in effect.

## DISCUSSION

This population-based prospective cohort study in Denmark revealed a significant association between neuroticism and the risk of death from all causes as well as from cancer-related death among persons diagnosed with cancer. Stratification by gender suggested that the effect was stronger among women compared with men, but no gender-dependent effect modification on neuroticism and risk of death was observed. No significant association was found between extroversion and risk of death from all causes nor of cancer-related death.

Five earlier studies investigated associations between personality traits using the Eysenck measurements and cancer survival. Nakaya *et al* (2005) followed up 890 cases of total cancer for 8 years and documented 356 deaths from all causes. No significant associations were found for extraversion, neuroticism, psychoticism, or lie. Ratcliffe *et al* (1995) followed-up 63 cases of Hodgkin's disease and non-Hodgkin's lymphoma for 5 years revealing 27 deaths from all causes. A positive significant, linear association was observed between lie and the risk of death from all causes (Linear trend *P*=0.001). Dean and Surtees (1989) followed up 121 postoperative cases of breast cancer for 6–8 years revealing 22 deaths from all causes. Extraversion, neuroticism, and lie showed no significant effect on cancer survival. Hislop *et al* (1987) followed-up 133 cases of breast cancer for 4 years revealing 26 deaths from all causes. A significant inverse association was observed between extraversion and the risk of death from all causes (multivariate HR=0.33, *P*<0.05). Neuroticism was not associated with risk of death (multivariate HR=0.85, *P*>0.05). Lastly, Greer *et al* (1979) followed-up 69 postoperative cases of breast cancer for 5 years revealing 18 deaths from all causes. No significant associations were found for extraversion and neuroticism. The results of the current study indicated that neuroticism was associated with cancer survival and this association was not observed in any of the previous studies, only associations between lie and extroversion and cancer survival was found (Hislop *et al*, 1987; Ratcliffe *et al*, 1995). The differences in the results found by the various studies could be a result of differences in methodological quality, or perhaps a chance finding.

An association between neuroticism and cancer survival is theoretically possible and could be related to stress. Studies have found that neuroticism leads to a high level of psychological distress (De Jong *et al*, 1999) and it is possible that stress, through a dysregulation of the immune system (Maddock and Pariante, 2001; Kiecolt-Glaser *et al*, 2002), could result in a poor cancer prognosis. It is also possible that the association is related to depression. In an earlier study using a population-based sample, neuroticism was reported to be a significant risk factor for major depression (Kendler *et al*, 2004). Furthermore, a number of prospective studies have reported a statistically significant association between depression (higher degree of depressive symptoms or psychiatrically diagnosed depression) and survival in patients with various types of cancer (Hjerl *et al*, 2003; Goodwin *et al*, 2004). The association between depression and cancer survival could be explained by potential intermediaries such as endocrinological or immunological pathways (Pettingale *et al*, 1981; Kiecolt-Glaser and Glaser, 1999; Spiegel and Kato, 1996), or by compliance with cancer treatment (Holland, 1987; Colleoni *et al*, 2000). Moreover, suicide is also thought to influence survival from cancer (Akechi *et al*, 2004; Yousaf *et al*, 2005).

This study had several methodological advantages compared with previous studies on personality and cancer survival. First, the study to the best of our knowledge included the second highest number of cancer cases and deaths in all of the studies conducted until now, and analyses were conducted on both death from all causes and cancer-related deaths. Both measures were important; ideally cancer-related death should be more accurate, but errors might be introduced at the assignment of cause of death and generally the autopsy rate is low; for example, in 1996 the overall autopsy rate in Denmark was 12.5% (Juel and Helweg-Larsen, 1999). All-cause of death is thus in comparison to cancer-related death a broader, but more clear and simple end point. Second, we controlled extensively for potential confounding variables, including cigarette smoking and alcohol consumption. A number of studies have reported that smoking and alcohol consumption are associated with extraversion and other personality traits (Arai *et al*, 1997) as well as with cancer survival (Tammemagi *et al*, 2004). Comparing point estimates of the HRs for neuroticism in the univariate and multivariate analyses, we observed differences suggesting that smoking and alcohol did affect cancer survival (Table 2). Also, personality traits were assessed before the cancer diagnosis, and to the best of our knowledge this has only been performed in one previous study (Nakaya *et al*, 2005). Assessing personality traits after the cancer diagnosis means that the assessment may have been influenced by the psychological reactions to the diagnosis or brought about by the treatment. To assess personality traits before the diagnosis may prevent this type of bias (Nakaya *et al*, 2003).

Our study also has some limitations. First, the number of persons diagnosed as having cancer in the current study population was small, especially among the men. One would prefer to stratify by cancer site since the treatment and prognosis differs for various cancer types. On the other hand, most of the studies, which in fact provided this kind of site-specific survival analyses were hampered by small numbers. The analyses may not have had sufficient statistical power to detect associations between small increases or decreases in the risk of death from all causes and personality traits. Also, the confidence limits in this study for the multivariable analyses on neuroticism among women were broad, and it is possible that the result was due to chance. Third, we had no information on health behaviours after the cancer diagnosis (Kuhn *et al*, 2005) or on compliance with treatment, which may have affected survival. Lastly, we lack a detailed theoretical mechanism explaining why the association between neuroticism and cancer survival was mainly present among women. It is possible that the mechanism is connected to depression. In the current study, the association between neuroticism and cancer survival was stronger among women. Also, the risk of depression is higher among women compared with men (Kendler *et al*, 2004). A potential explanation for the observed stronger association between neuroticism and cancer survival among women could thus be that high neuroticism involves an increased risk of depression, which may affect the risk of death. Evidence supporting this hypothesis in the current study is weak since we found no gender-related effect modification and no deaths were related to suicide. It would be highly relevant for future research to include measures of depression in order to shed light on whether depression can act as a mediating factor.

In conclusion, the results from this population-based prospective cohort study in Denmark support the hypothesis that neuroticism is positively associated with cancer survival. Stratification by sex suggested that the effect was stronger among women compared with men, but no gender-related significant effect modification on neuroticism and risk of all cause or cancer-related death was observed. Further research on neuroticism and cancer survival within specific cancer types and including a large population is needed.

## Figures and Tables

**Table 1:** Demographic and medical characteristics according to extraversion and neuroticism in 189 persons diagnosed with cancer

	**Extraversion**				**Neuroticism**			
**Characteristics**	**⩽3**	**4–6**	**⩾7**	***P*-value**	**⩽2**	**3–4**	**⩾5**	***P*-value**
No. of subjects	59	77	53		81	59	49	
Age in years at cancer diagnosis, mean±s.d.	55±8	54±8	56±7	0.38	55±8	55±8	54±8	0.88
								
*Sex* (%)				<0.01				0.03
Man	20	33	53		44	31	23	
Woman	80	68	47		56	70	78	
								
*Cancer type* (%)				0.72				
Digestive organs (excluding liver)	12	10	6		9	9	12	0.91
Hormone-related organs^a^	24	21	13		20	17	23	
Virus and immune-related organs^b^	44	43	47		42	53	39	
Respiratory organs	10	10	13		14	9	10	
Other organs	10	16	21		16	14	16	
								
*Clinical stage* (%)				0.69				0.11
*In situ*	14	16	9		11	17	12	
Localised	42	40	47		37	46	49	
Regional invasion	3	10	13		15	3	6	
Distant metastasis	17	13	13		20	14	6	
Unknown	24	21	17		17	20	27	
								
*Length of education in years* (%)				0.60				0.71
<10	75	71	79		72	76	78	
⩾10	25	29	21		28	24	23	
								
*Marital status, living with spouse* (%)				0.83				0.01
Yes	85	88	87		88	95	76	
No	15	12	13		12	8	25	
								
*Social class*^c^ (%)				0.12				0.07
I, II	19	16	28		27	19	10	
III, IV	28	58	57		51	61	53	
V	34	26	15		22	20	37	
								
*Smoking status at baseline* (%)				0.02				0.48
Never smokers	46	26	23		35	25	33	
Past smokers	15	10	9		15	10	8	
Current smokers	39	64	68		51	64	59	
								
*Alcohol consumption at baseline (g week*^−*1*^) (%)				<0.01				0.21
0	31	21	13		19	24	24	
1–150	58	64	51		60	49	65	
⩾151	12	16	36		21	27	10	
								
*Psychiatric status* d								<0.01
Normal	81	82	89	0.70	96	90	55	
Neurotic	15	16	8		1	8	39	
Deviant/psychotic	3	3	4		2	2	6	

a Including cancers of the breast, corpus uteri, ovary, and prostate.

b Including cancers of the skin, non-Hodgkin's lymphoma, Hodgkin's disease, leukaemia, liver cancer, and cancer of the cervix uteri.

c The social class variable is based on a classification constructed by the Social Research Institute in Denmark and is based on a combination of information on ownership of land, education, employment status, and number of subordinates. Social group I is the highest social group.

d As evaluated by the physician who conducted the social-psychiatric interview.

**Table 2:** Hazard ratios (HRs) for death from all causes and 95% confidence intervals (CIs) according to demographic and medical characteristics in 189 persons diagnosed with cancer

				**Univariate analysis**	
**Characteristics**	**No. of subjects**	**Median survival years**	**No. of cases**	**HR (95%CI)^a^**	***P*-value**
*Age in years at cancer diagnosis*					
Continuous variable	189	6	82	1.05 (1.02–1.08)	<0.01
					
*Sex*					
Man	65	3	37	2.0 (1.3–3.0)	<0.01
Woman	124	9	45	1.0 (referent)	
					
*Cancer type* (%)					
Digestive organs (excluding liver)	18	4	9	1.0 (referent)	
Hormone-related organs^b^	37	5	15	0.7 (0.3–1.5)	0.33
Virus and immune-related organs^c^	84	12	21	0.4 (0.2–0.8)	0.01
Respiratory organs	21	1	18	3.7 (1.7–8.4)	<0.01
Other organs	29	3	19	1.5 (0.7–3.3)	0.31
					
*Clinical stage* (%)					
*In situ*	25	26	3	1.0 (referent)	
Localised	81	7	24	4.1 (1.2–14.2)	0.02
Regional invasion	17	1	11	18.2 (4.9–68.1)	<0.01
Distant metastasis	27	0	27	52.5 (14.9–185.1)	<0.01
Unknown	39	6	17	6.7 (1.9–23.9)	<0.01
					
*Educational periods in years* (%)					
<10	141	6	65	1.3 (0.8–2.3)	0.28
⩾10	48	7	17	1.0 (referent)	
					
*Marital status, living with spouse* (%)					
Yes	164	6	71	1.0 (referent)	
No	25	5	11	0.9 (0.5–1.6)	0.64
					
*Social class* (%)					
I, II	38	4	15	1.0 (referent)	
III, IV	103	7	44	1.0 (0.6–1.9)	0.90
V	48	7	23	1.2 (0.6–2.3)	0.57
					
*Smoking status at baseline* (%)					
Never smokers	59	9	15	1.0 (referent)	
Past smokers	22	4	11	2.4 (1.1–5.3)	0.02
Current smokers	108	4	56	2.6 (1.4–4.5)	<0.01
					
*Alcohol consumption at baseline (g week*^−*1*^) (%)					
0	41	7	15	1.0 (referent)	
1–150	110	7	44	1.1 (0.6–1.9)	0.82
⩾151	38	3	23	2.0 (1.0–3.8)	0.04
					
*Psychiatric status* d					
Normal	158	6	65	1.0 (referent)	
Neurotic	25	3	15	1.7 (1.0–3.0)	0.053
Deviant/psychotic	6	6	2	0.7 (0.2–2.9)	0.64

a All hazard ratios (HRs) are given with 95% confidence intervals (CIs) in parentheses.

b Including cancers of the breast, corpus uteri, ovary, and prostate.

c Including cancers of the skin, non-Hodgkin's lymphoma, Hodgkin's disease, leukaemia, liver cancer, and cancer of the cervix uteri.

d As evaluated by the physician who conducted the social-psychiatric interview.

**Table 3:** Hazard ratios (HRs) and 95% confidence intervals (CIs) for risk of all-cause death according to extraversion and neuroticism in 189 persons diagnosed with cancer

	**Extraversion**			**Linear trend test^b^**		**Neuroticism**			**Linear trend test^a^**	
	**⩽3**	**4–6**	**⩾7**	**Linear**	***P*-value**	**⩽2**	**3–4**	**⩾5**	**Linear**	***P*-value**
*Total subjects*										
No. of death/no. of subjects	27/59	29/77	26/53			35/81	22/59	25/49		
Median survival years	4	9	5			5	9	5		
Age and sex adjusted HR	1.0 (referent)	0.7 (0.4–1.2)	1.0 (0.5–1.8)	1.0 (0.9–1.1)	0.61	1.0 (referent)	0.9 (05–1.6)	1.5 (0.9–2.6)	1.1 (1.0–1.2)	0.12
Multivariable adjusted HR1	1.0 (referent)	0.5 (0.3–1.0)	0.8 (0.4–1.7)	0.9 (0.8–1.1)	0.33	1.0 (referent)	1.2 (0.6–2.1)	2.6 (1.4–5.0)	1.2 (1.1–1.4)	<0.01
Multivariable adjusted HR2	1.0 (referent)	0.6 (0.3–1.0)	0.9 (0.4–1.7)	0.9 (0.8–1.1)	0.34	1.0 (referent)	1.1 (0.6–2.1)	2.3 (1.1–4.7)	1.2 (1.0–1.3)	0.04
Multivariable adjusted HR3	1.0 (referent)	0.6 (0.3–1.1)	0.8 (0.4–1.7)	1.0 (0.8–1.1)	0.38	1.0 (referent)	1.2 (0.6–2.2)	2.6 (1.2–5.4)	1.2 (1.0–1.3)	0.02
Multivariable adjusted HR4	1.0 (referent)	0.5 (0.2–0.9)	0.6 (0.3–1.3)	0.9 (0.8–1.0)	0.12	1.0 (referent)	0.9 (0.4–1.7)	2.2 (1.0–4.8)	1.2 (1.0–1.4)	0.04
										
*Men*										
No of death/total	8/12	13/25	16/28			19/36	11/18	7/11		
Median survival years	2	5	4			4	3	3		
Multivariable adjusted HR1	1.0 (referent)	1.3 (0.4–4.2)	1.2 (0.4–4.0)	1.0 (0.8–1.2_	0.99	1.0 (referent)	1.6 (0.6–4.3)	2.2 (0.6–4.3)	1.2 (1.0–1.2)	0.08
Multivariable adjusted HR2	1.0 (referent)	1.1 (0.3–3.9)	1.2 (0.4–4.1)	1.0 (0.8–1.2)	0.98	1.0 (referent)	1.4 (0.5–4.3)	1.8 (0.5–7.1)	1.2 (0.9–1.5)	0.17
										
*Women*										
No of death/total	19/47	16/52	10/25			16/45	11/41	18/38		
Median survival years	7	11	9			9	9	7		
Multivariable adjusted HR1	1.0 (referent)	0.5 (0.2–1.2)	0.7 (0.3–1.9)	0.9 (0.8–1.1)	0.18	1.0 (referent)	0.7 (0.3–2.0)	3.4 (1.4–8.6)	1.3 (1.1–1.5)	<0.01
MultivariableHR2	1.0 (referent)	0.5 (0.2–1.2)	0.7 (0.3–1.9)	0.9 (0.8–1.1)	0.18	1.0 (referent)	0.7 (0.2–1.9)	3.2 (1.1–8.7)	1.2 (1.0–1.6)	0.03

Multivariable HR1 was adjusted for age at cancer diagnosis (continuous variable), sex, cancer type (digestive organs, hormone-related organs, virus and immune-related organs, respiratory organs, or other organs), clinical stage (*in situ*, localised, regional invasion, distant metastasis, or unknown), smoking status at baseline (never smoker, past smoker, or current smoker), and alcohol consumption in grams per week at baseline (0, 1–150, or 151 or more).

Multivariable HR2 was adjusted for psychiatric status (normal, neurotic, or deviant/psychotic) in addition to variables adjusted for using multivariable HR1.

HR3 was estimated excluding 19 subjects who were diagnosed with cancer within the first 3 years from baseline and was adjusted for using the same variables as in multivariable HR2.

HR4 was estimated with cancer mortality as the end point and was adjusted for using the same variables as in multivariable HR2.

a Linear trend tests were calculated by treating the personality subscales as continuous variables.
