# Personality traits and cancer survival: a Danish cohort study

**DOI:** 10.1038/sj.bjc.6603489

**Published:** 2006-11-28

**Authors:** N Nakaya, P E Hansen, I R Schapiro, L F Eplov, K Saito-Nakaya, Y Uchitomi, C Johansen

**Correction to**: *British Journal of Cancer* (2006) **95**, 146–152. doi:10.1038/sj.bjc.6603244

Owing to an author error, a wrong statement regarding the interpretation of the results on neuroticism was included in the abstract and in the conclusion. The data themselves are correct, but instead of stating that ‘This study showed that neuroticism is positively associated with cancer survival’ it should have stated that ‘This study showed that neuroticism is negatively associated with cancer survival’. The authors would like to point out that, although they do not think that this represents a major flaw – they would, nevertheless, prefer to have it corrected.

